# A Hybrid IMRT/VMAT Technique for the Treatment of Nasopharyngeal Cancer

**DOI:** 10.1155/2015/940102

**Published:** 2015-01-21

**Authors:** Nan Zhao, Ruijie Yang, Yuliang Jiang, Suqing Tian, Fuxin Guo, Junjie Wang

**Affiliations:** Department of Radiation Oncology, Peking University Third Hospital, Beijing 100191, China

## Abstract

Hybrid IMRT/VMAT technique which combined intensity modulated radiotherapy (IMRT) and volumetric modulated arc therapy (VMAT) was developed for the treatment of nasopharyngeal cancer (NPC). Two-full-arc VMAT (2ARC-VMAT), 9-field IMRT (9F-IMRT), and Hybrid IMRT/VMAT plans for NPC were compared in terms of the dosimetric quality, sparing of organs at risk (OARs), and delivery efficiency. The Hybrid IMRT/VMAT technique can improve the target dose homogeneity and conformity compared with 9F-IMRT and 2ARC-VMAT. It can reduce the dose delivered to the TMJ, mandible, temporal lobe, and unspecified tissue with fewer MUs compared with 9F-IMRT and dose delivered to parotids, brainstem, and spinal cord compared with 2ARC-VMAT technique. The mean delivery time of Hybrid plans was shorter than that of 9F-IMRT plans (408 s versus 812 s; *P* = 0.00) and longer than that of 2ARC-VMAT plans (408 s versus 179 s; *P* = 0.00). Hybrid IMRT/VMAT technique could be a viable radiotherapy technique with better plan quality.

## 1. Introduction

Nasopharyngeal cancer (NPC) remains one of the highest incident cancers in southern China and East Asia. Three-dimensional conformal radiotherapy (3D-CRT) was used to deliver a more conformal dose distribution compared with conventional radiotherapy for the radiotherapy of NPC. Although the conformity of dose distribution improved with 3D-CRT, it cannot achieve a satisfactory normal tissue sparing [1]. Intensity-modulated radiation therapy (IMRT) can provide better parotid sparing and improved quality of life compared with three-dimensional conformal radiotherapy (3D-CRT) for early-stage NPC [2]. However, although IMRT has become a common technique for NPC radiotherapy, the concern regarding its high number of monitor units (MUs) and long treatment time is still under discussion [3]. High number of MUs can increase collimator transmission and scatter radiation from the linac which can increase the risk of secondary tumors [4]. Volumetric modulated arc therapy (VMAT) has more flexibility of dose delivery through a full range of angles (gantry rotation) with continuous modulation of beam aperture and variable dose rate [5, 6]. There have been many studies demonstrating that VMAT offers equivalent or superior target coverage and greatly improves organs at risk (OARs) sparing with much lower MUs and shorter treatment time [3, 7, 8]. However, there were some contradictory conclusions regarding the dosimetric comparison between VMAT and IMRT. Ning et al. [9] found that target dose homogeneity and conformity for single arc VMAT (VMAT1) and double arc VMAT (VMAT2) were superior to those for 9-field IMRT, whereas the mean dose of the parotid gland for 9-field IMRT was significantly reduced compared to those for VMAT1 and VMAT2. Jin et al. [10] reported that two-arc whole-field simultaneous integrated boost VMAT achieved superior target coverage compared with one-arc whole-field simultaneous integrated boost VMAT and similar target coverage compared with 7-field IMRT. No significant differences of OARs sparing were found between VMAT and IMRT.

Hybrid technique combining IMRT and VMAT has the potential benefit to improve the dose distribution compared with IMRT and VMAT. The reason was that IMRT and VMAT made compromises in different aspects. IMRT achieved a reasonable dose distribution by intensity modulation with limited angular beam sampling. Due to the sparse angular sampling in IMRT, the conformity of the resultant dose distribution was often limited. On the other hand, while VMAT had sufficient angular sampling, it did not provide the desired intensity modulation in some beam directions. The final dose distribution depended on the level of intensity modulation and angular sampling. Hybrid technique can improve the dose distribution by increasing the freedom to find the optimal combination of angular sampling and intensity modulation.

The aim of this study is to investigate a radiotherapy technique that is called Hybrid IMRT/VMAT which has been designed to combine 7-field IMRT and one-arc VMAT to find out if it can improve the target coverage and OARs sparing for the patients with nasopharyngeal carcinoma. The dosimetric quality and delivery efficiency of the Hybrid IMRT/VMAT technique were evaluated by comparing with IMRT and VMAT.

## 2. Materials and Methods

### 2.1. Patients' Characteristics

10 patients with NPC who had undergone radiotherapy continuously from February 2013 to June 2014 in our hospital were retrospectively selected for this study. Eight cases were American Joint Committee on Cancer (AJCC) stage III or IV and two cases were AJCC IIA (T_2a_N_0_M_0_) nasopharyngeal carcinoma.

### 2.2. Delineation of Target Volumes and Critical Structures

The patients underwent computed tomography (CT) scanning in 3 mm slice thickness in supine arm-up position. Gross tumor volume (GTV) was defined as the visualization of any gross tumor and abnormal lymph nodes as seen on CT images or other images (magnetic resonance imaging and positron emission tomography). Clinical target volume (CTV) was defined as the GTV plus areas considered at risk for containing microscopic disease delineated by the treating physician. CTV_70_ was defined as GTV plus a margin of 5 mm around the GTV. This margin can be reduced to as low as 1 mm for tumors in close proximity to critical structures. For CTV_59.4_, all potential routes of spread for primary and nodal GTVs should be delineated by the treating radiation oncologist. The low anterior neck is defined as a low risk subclinical region, with the prescription dose of 54 Gy (CTV_54_). A margin of 3 mm was added to all volumes of CTVs to create the respective PTVs. The PTVs were trimmed to 3 mm from the skin surface. The OARs delineated included the brainstem, spinal cord, optic nerves, optic chiasm, parotid glands, temporomandibular joints (TMJ), mandible, eyes, lens, temporal lobes, and unspecified tissue. The unspecified tissue is defined as the tissue within the skin surface and outside all other critical normal structures and PTVs. The spinal cord was contoured starting at least 2 cm above the superior extent of the PTV and continuing on every CT slice to at least 2 cm below the inferior extent of the PTV.

### 2.3. Treatment Planning

Hybrid IMRT/VMAT, IMRT, and VMAT plans were designed for each patient. Dose prescription included 70 Gy to PTV_70_, 59.4 Gy to PTV_59.4_, and 54 Gy to PTV_54_ with the plan normalization to cover 95% of the PTV_54_ with 100% of the prescribed dose. Eclipse 10.0 (Varian, Palo Alto, CA) treatment planning system was used for treatment planning, utilizing 6 MV photon beams generated from Varian Trilogy linac equipped with a 120-leaf Millennium Multileaf Collimator (MLC).

### 2.4. IMRT

The IMRT plans were generated with nine coplanar fields with equal-spaced gantry angles (9F-IMRT). 9F-IMRT plan optimization was performed by utilization of the Dose Volume Optimizer (Varian Eclipse version 10.0) algorithm in the Eclipse treatment planning system. The plans were iteratively optimized to obtain the optimal PTV coverage and OARs sparing. After inverse planning, the leaf sequences using sliding window technique were generated for 9F-IMRT plans.

### 2.5. VMAT

A VMAT double-arc plan (2ARC-VMAT) was optimized using the progressive resolution optimization in the Eclipse treatment planning system (version 10.0). The optimization objectives of the 2ARC-VMAT plans were the same with the 9F-IMRT plans. To minimize the leakage, tongue, and groove effects, the collimator angle varied between 0° and 90° according to the shape of the target. Other planning parameters were MLC motion speed 0 to 2.5 cm/s, gantry rotation speed 0.5 to 4.8 degrees/s, and dose rate 0 to 600 MU/min. There are 354 control points in the double rotational arcs. The control points described gantry speed, dose rate, total delivery time, and leaf travel speed.

### 2.6. Hybrid IMRT/VMAT

The Hybrid IMRT/VMAT plans were combination of 1-full-arc VMAT (Hybrid-VMAT) and 7-field IMRT (Hybrid-IMRT). The Hybrid-IMRT plans delivered half of the prescribed dose while the Hybrid-VMAT parts consisted of one full arc which was optimized with the Hybrid-IMRT plan as a base plan, to deliver the other half prescribed dose. The beam angles of Hybrid-IMRT were initially optimized by the beam angle optimization (BAO) algorithm (Varian Eclipse 10.0). The number of the fields was confined to seven. Some beam angles were adjusted according to the experience of the dosimetrist, if the results of the BAO did not satisfy the dosimetric criteria. The same optimization objectives and planning parameters were used for the Hybrid IMRT/VMAT, 2ARC-VMAT, and 9F-IMRT plans.

### 2.7. Dosimetric Evaluation

Dosimetric quality of plans was evaluated by means of dose-volume histogram (DVH) for all three techniques. To evaluate the dose distribution of the target, parameters were calculated for all the three PTVs: minimal dose delivered to the 98% of the target volume (*D*_98%_), the maximum dose delivered to the 2% of the target volume (*D*_2%_), conformation number (CN), and homogeneity index (HI) according to the ICRU report 83 [11]. The CN was defined using the equation [12]:

(1) $$\begin{array}{c} \text{CN}=\frac{\text{T}{\text{V}}_{\text{RI}}}{\text{TV}}\times \frac{\text{T}{\text{V}}_{\text{RI}}}{V_{\text{RI}}}, \end{array}$$

where CN = conformation number, TV_RI_ = target volume covered by the reference isodose, TV = target volume, and *V*_RI_ = volume of the reference isodose. The CN ranged from 0 to 1, where 1 was the ideal value. A larger CN indicated a smaller volume of the prescription dose delivered outside the PTV. The HI was defined using the following equation [11]:

(2) $$\begin{array}{c} \text{HI}=\frac{D_{2\%}-D_{98\%}}{D_{50\%}}, \end{array}$$

where *D*_2%_ meant near-maximum dose, *D*_98%_ meant near-minimum dose, and *D*_50%_ meant the dose that half volume of the PTV received. An HI of 0 indicated that the absorbed-dose distribution was almost homogenous. A larger HI indicated a greater dose exceeding the prescribed dose and/or a larger volume of the target receiving too small dose. The evaluation criteria of OARs were defined basically according to RTOG 0615 protocols. Mean dose and *V*_30_ were recorded and compared for both left and right parotids, as well as the maximum dose of the spinal cord, brainstem, optic nerve, optic chiasm, double lens, and double eyes. The near maximum dose (*D*_2%_) of double TMJ, mandible, and double temporal lobes was also compared.

### 2.8. Treatment Delivery Time and MUs

The Hybrid IMRT/VMAT, 9F-IMRT, and 2ARC-VMAT plans for 10 patients were delivered to a solid water phantom (Multicube Phantom, IBA, Germany) on the Trilogy linear accelerator. The treatment delivery time and MUs were recorded and evaluated. The treatment delivery time was defined as the time from the first beam-on until the last beam is turned off.

### 2.9. Statistical Analysis

Repeated measures ANOVA were used to compare the three techniques. Statistical analysis was performed using the IBM SPSS Statistics (version 21.0, New York, USA) for Windows. Differences were reported to be statistically significant at *P* < 0.05.

## 3. Results

**Figure 1:**
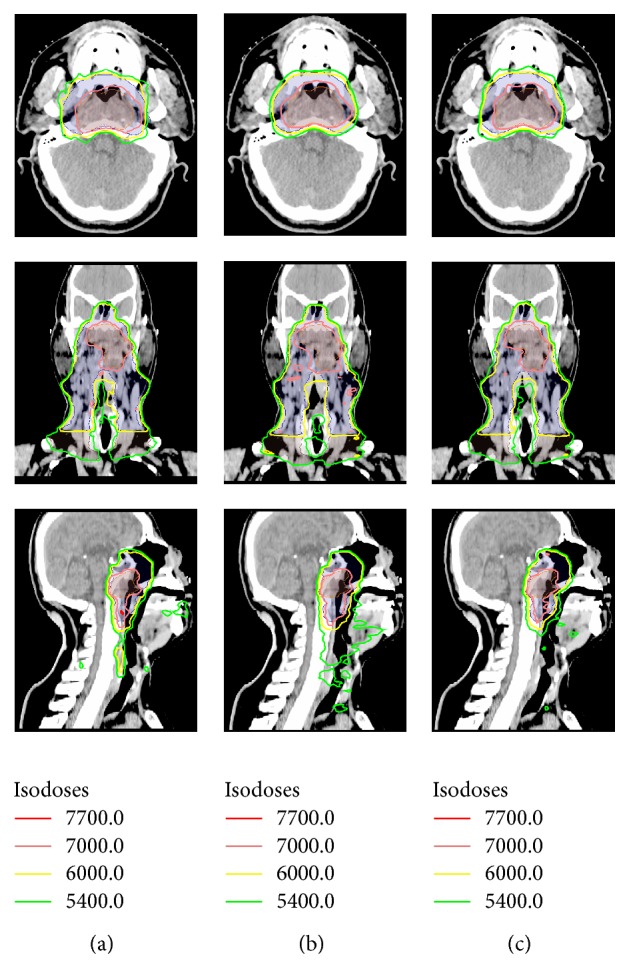
Dose distributions on axial, coronal, and sagittal views for one representative case: (a) 9F-IMRT, (b) 2ARC-VMAT, and (c) Hybrid IMRT/VMAT.

**Figure 2:**
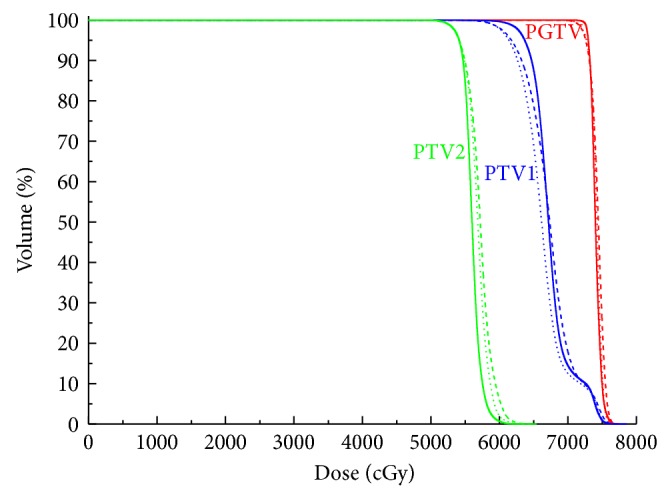
Representative dose-volume histogram of PTVs for 9F-IMRT, 2ARC-VMAT, and Hybrid IMRT/VMAT. The curves of 9F-IMRT, 2ARC-VMAT, and Hybrid IMRT/VMAT are indicated in solid lines, dashed lines, and dotted lines, respectively.

**Figure 3:**
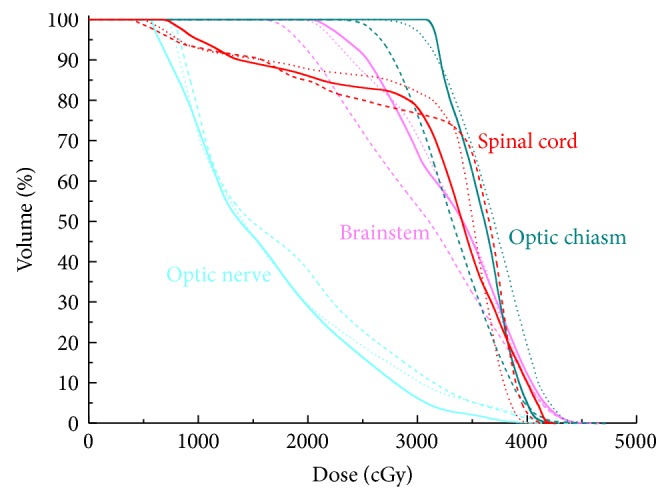
Representative dose-volume histogram of spinal cord, optic chiasm, brainstem, and optic nerve for 9F-IMRT, 2ARC-VMAT, and Hybrid IMRT/VMAT. The curves of 9F-IMRT, 2ARC-VMAT, and Hybrid IMRT/VMAT are indicated in solid lines, dashed lines, and dotted lines, respectively.

**Figure 4:**
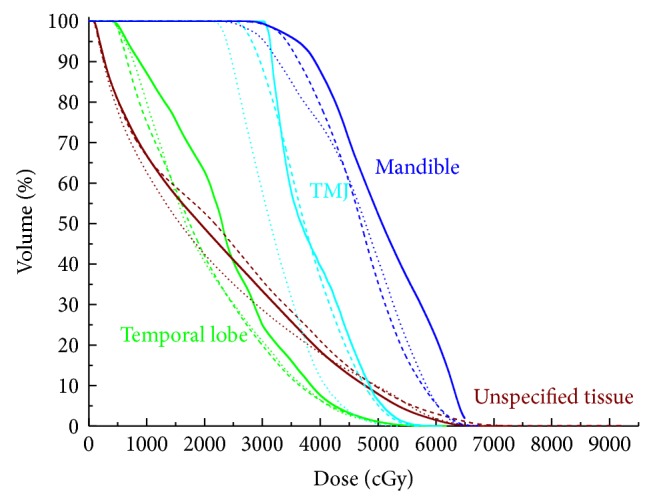
Representative dose-volume histogram of mandible, TMJ, temporal lobe, and unspecified tissue for 9F-IMRT, 2ARC-VMAT, and Hybrid IMRT/VMAT. The curves of 9F-IMRT, 2ARC-VMAT, and Hybrid IMRT/VMAT are indicated in solid lines, dashed lines, and dotted lines, respectively.

The mean volumes of the PTV_70_, PTV_59.4_, and PTV_54_ were 82.11 cm^3^ (27.84 cm^3^ to 196.64 cm^3^), 445.10 cm^3^ (209.40 cm^3^ to 777.80 cm^3^), and 264.01 cm^3^ (121.78 cm^3^ to 549.21 cm^3^), respectively. For all 10 cases, all the plans were clinically acceptable, with at least 98% PTV receiving 95% of the prescribed dose. The typical isodose distribution and DVH comparison were given from Figures 1, 2, 3, and 4.

### 3.1. Target Coverage

Dose homogeneity and conformity of 9F-IMRT, 2ARC-VMAT, and Hybrid IMRT/VMAT plans for each PTV were shown in Table 1. For PTV_59.4_, the Hybrid IMRT/VMAT and 9F-IMRT techniques significantly improved target dose homogeneity compared with 2ARC-VMAT (*P* = 0.01; *P* = 0.00). For PTV_54_, the Hybrid IMRT/VMAT and 9F-IMRT techniques significantly improved target dose homogeneity compared with 2ARC-VMAT (*P* = 0.00; *P* = 0.04). For PTV_70_, the Hybrid IMRT/VMAT technique significantly improved target dose homogeneity compared with 2ARC-VMAT (*P* = 0.05). For PTV_70_ and PTV_59.4_, the Hybrid IMRT/VMAT technique significantly improved target dose conformity compared with 9F-IMRT (0.62 versus 0.47, *P* = 0.01; 0.64 versus 0.58, *P* = 0.01) and 2ARC-VMAT (0.62 versus 0.43, *P* = 0.00; 0.64 versus 0.60, *P* = 0.01). For PTV_54_, the Hybrid IMRT/VMAT technique improved target dose conformity compared with 9F-IMRT (0.69 versus 0.63; *P* = 0.00).

### 3.2. Organs at Risk Sparing

The mean values of the OARs from the DVH parameters were listed in Table 2. The near maximum dose (*D*_2%_) and mean dose of mandible in Hybrid IMRT/VMAT were 5.2% (*P* = 0.00) and 4.2% (*P* = 0.03) lower than in 9F-IMRT plans, respectively. The mean dose of TMJ, temporal lobe, and unspecified tissue for Hybrid plans was 12.8% (*P* = 0.00), 11.4% (*P* = 0.01), and 4.0% (*P* = 0.02) lower than 9F-IMRT plans, respectively. The* D*_1%_ of unspecified tissue for Hybrid plans was 61.1 Gy, with an absolute difference of 3.6 Gy lower than 9F-IMRT plans (*P* = 0.00). The* V*_30_ of right and left parotids for Hybrid plans and VMAT plans were 34.7% versus 35.7% (*P* = 1.00) and 36.1% versus 37.1% (*P* = 1.00), respectively. The mean dose of TMJ and* D*_2%_ of mandible for Hybrid plans and 2ARC-VMAT plans were 37.4 Gy versus 39.3 Gy (*P* = 0.34) and 63.7 Gy versus 64.7 Gy (*P* = 0.06), respectively. Though there were no significant differences in the *D*_max⁡_ of brainstem and the *D*_0.03cc_ of spinal cord among the three techniques, the *D*_max⁡_ of brainstem for Hybrid plans was lower than that for 9F-IMRT (*P* = 1.00) and 2ARC-VMAT (*P* = 1.00) plans and the *D*_0.03cc_ of spinal cord for Hybrid plans was lower than that for 2ARC-VMAT plans (*P* = 0.61).

### 3.3. Treatment Delivery Time and MUs

The treatment delivery time and MUs for three techniques were summarized in Table 3. The mean delivery time of Hybrid plans was shorter than that of 9F-IMRT plans (408 s versus 812 s; *P* = 0.00) and longer than that of 2ARC-VMAT plans (408 s versus 179 s; *P* = 0.00). The mean MUs of Hybrid plans were between the values of 9F-IMRT and 2ARC-VMAT plans.

## 4. Discussion

In this study, a Hybrid IMRT/VMAT technique was developed based on Hybrid-IMRT and Hybrid-VMAT for NPC. For PTV_59.4_ and PTV_54_, the Hybrid IMRT/VMAT and 9F-IMRT techniques significantly improved target dose homogeneity compared with 2ARC-VMAT. Johnston et al. [13] made a comparison between VMAT plans and IMRT plans for ten patients with locoregionally advanced oropharynx or nasopharynx carcinoma. They found that the target dose homogeneity in IMRT was better than that in VMAT plans for PTV_63_ and PTV_56_. Except for PTV_54_, the Hybrid IMRT/VMAT technique significantly improved target dose conformity compared with 9F-IMRT and 2ARC-VMAT. The improvement of conformal dose distribution was especially important when the targets were in close proximity to the critical organs. The more homogenous dose distribution can improve the target coverage and the tumor control [14].

The Hybrid technique significantly reduced the* D*_2%_ of mandible, mean dose of mandible, TMJ, and temporal lobe compared with 9F-IMRT. The mean dose and* D*_2%_ of TMJ for Hybrid plans were lower than 2ARC-VMAT although there were no significant differences. There have been some studies demonstrating that radiotherapy to TMJs can result in limitations in mouth opening [15]. The loss of function and range of mandibular motion seemed to be correlated with the fibrosis in the muscles of mastication and necrosis of soft tissues and bone [16].

Although there were no significant differences, the mean doses of right and left parotids were lower for Hybrid plans compared with 9F-IMRT. The* V*_30_ of right and left parotids were lower for Hybrid plans compared with 2ARC-VMAT. Grégoire et al. [17] had proved that the mean doses of the parotids were related to their residual salivary output. Münter et al. [18] and Li et al. [19] found that the recovery was substantial if the parotid gland doses were lower than 25–30 Gy and function may return to pretreatment levels 2 years after radiotherapy. Scorsetti et al. [20] reported side effects that 45 patients after radiotherapy had: 28% of the patients experienced G3 mucositis, 14% G3 dermatitis, and 44% G2 dysphagia (the mean dose of parotid < 26 Gy). Therefore, it was beneficial to lower the mean dose of the parotids.

There have been some studies demonstrating that single-arc VMAT can achieve superior or equivalent plan quality in simple target such as prostate cancer compared with IMRT [21]. As the complexity of target increased, single-arc VMAT was inferior to IMRT in target coverage for head and neck cancer, whereas double-arc VMAT was superior in target coverage and OARs sparing compared with IMRT [3]. Due to the complexity of the target, 9-field IMRT was used to treat the patients. Kan et al. [22] evaluated the 9-field IMRT, double-arc VMAT (RA2), and triple-arc (two full arcs plus one partial arc) VMAT (RA3) on different geometric complexity targets for NPC. They found that RA2 plans were slightly inferior to IMRT and RA3 plans for most cases. However, 9-field IMRT has longer treatment time and more MUs which can increase the risk of target intrafraction movements and the possibility of secondary radiation-induced tumor. So, a technique which combined the single arc and 7-field IMRT was developed to enhance the efficiency and improve the quality of the plans.

As expected, the required MUs for Hybrid plans were somewhere between values for 2ARC-VMAT and 9F-IMRT. There appeared to be two factors affecting the required MUs for Hybrid IMRT/VMAT. First, the Hybrid-IMRT component of Hybrid IMRT/VMAT, and particularly the complexity of MLC sequences, affected the required MUs significantly. The second factor affecting required MUs was the average field size of optimized Hybrid-VMAT apertures. In cases where the target volumes were surrounded by OARs with demanding optimization constraints, apertures would contain increased blocking of OARs, and more MUs would be required to deliver a specified dose to the PTV. In contrast, a plan with more open apertures would approach the comparatively efficient VMAT scenario with respect to MUs required. The relative longer delivery time for Hybrid plans was due to the 7-field IMRT part of Hybrid IMRT/VMAT with longer setup and gantry rotation time.

Additional research work on the Hybrid IMRT/VMAT strategy is warranted in several areas. Firstly, the prescription dose ratio between Hybrid-IMRT and Hybrid-VMAT in Hybrid IMRT/VMAT influences the dose distribution and delivery efficiency. The prescription dose ratio between Hybrid-IMRT and Hybrid-VMAT in Hybrid IMRT/VMAT was 1 : 1 in this study. Hybrid-IMRT was used as the base plan when optimizing the Hybrid-VMAT plan to achieve trade-off between better dosimetric quality and delivery efficiency. Secondly, the ideal orientation and number of Hybrid-IMRT beams, the number of Hybrid-VMAT arcs, and the start and stop angle of arcs in hybrid plans would likely vary for different sites. For the Hybrid-VMAT plan, one full arc with the gantry angle 181° to 179° was used in this study. For Hybrid-IMRT plan, manual beam selection is influenced by the nonintuitive dose contribution of each beam to the target and critical normal tissues. Selecting several beam angles by planners from a large number of beams with different orientations may increase quality assurance time, planning time, and treatment time [23]. In this study, the beam angles of the Hybrid-IMRT plan were initially optimized using the beam angle optimization (BAO) algorithm. BAO used automated computational schemes to reduce the number of beams and simultaneously improved the quality of IMRT plans [24, 25]. An optimization algorithm was needed to optimize both Hybrid-VMAT and Hybrid-IMRT simultaneously to determine the optimal proportion of the prescribed dose for the Hybrid-IMRT and Hybrid-VMAT components, the delivery sequence integrating the Hybrid-IMRT and Hybrid-VMAT components. So that the full potential of hybrid technique can be explored, the hybrid plans can be planned and delivered together, not separately. The Hybrid IMRT/VMAT technique can be implemented to find the optimal compromise between gantry angle and intensity modulation degrees of freedom, dosimetric quality, and delivery efficiency. It may be delivered without switching between delivery techniques in the future. That is, hybrid plans will be delivered as modulated arcs with IMRT inside, that is, IMRT control points (with no gantry motion) within a VMAT control point sequence (with gantry changes) rather than current two separate components, so that the delivery time would be further reduced. In addition, the emergence of auto field sequencing which eliminates the unnecessary operator manual control of gantry rotation during dose delivery and the dramatically increased dose rate in modern digital LINACs will make Hybrid IMRT/VMAT more efficient. Furthermore, the types of cancer sites and geometries that will benefit most from this Hybrid IMRT/VMAT technique should be further investigated.

## 5. Conclusions

Hybrid IMRT/VMAT technique can improve the target dose homogeneity and conformity compared with 2ARC-VMAT and can improve the target dose conformity compared with 9F-IMRT. Hybrid IMRT/VMAT technique reduced the dose of TMJ, mandible, temporal lobe, and unspecified tissue compared with 9F-IMRT with fewer MUs and reduced the dose of* V*_30_ of both parotids, brainstem, and spinal cord compared with VMAT.

## Figures and Tables

**Table 1:** Dosimetric comparison of PTV for IMRT, VMAT, and Hybrid IMRT/VMAT plans.

	9F-IMRT	2ARC-VMAT	Hybrid	Pairwise comparison		
				*p*_*α*_	*p*_*β*_	*p*_*γ*_
HI						
PTV_70_	0.07 ± 0.03	0.08 ± 0.03	0.06 ± 0.03	0.32	0.89	0.05
PTV_59.4_	0.16 ± 0.04	0.20 ± 0.04	0.17 ± 0.04	0.00	0.92	0.01
PTV_54_	0.12 ± 0.02	0.14 ± 0.02	0.11 ± 0.02	0.04	0.00	0.00
CN						
PTV_70_	0.47 ± 0.18	0.43 ± 0.12	0.62 ± 0.09	1.00	0.01	0.00
PTV_59.4_	0.58 ± 0.09	0.60 ± 0.10	0.64 ± 0.09	0.37	0.01	0.01
PTV_54_	0.63 ± 0.04	0.67 ± 0.05	0.69 ± 0.04	0.10	0.00	0.66

HI = homogeneity index; CN = conformation number.

*p*_*α*_: 9F-IMRT versus 2ARC-VMAT; *p*_*β*_: 9F-IMRT versus Hybrid; *p*_*γ*_: 2ARC-VMAT versus Hybrid.

**Table 2:** Dosimetric comparison of OARs for 9F-IMRT, 2ARC-VMAT, and Hybrid IMRT/VMAT plans.

	9F-IMRT	2ARC-VMAT	Hybrid	Pairwise comparison		
				*p*_*α*_	*p*_*β*_	*p*_*γ*_
Spinal cord						
*D*_max⁡_ (0.03 cc) (Gy)	41.3 ± 1.9	42.3 ± 3.1	41.7 ± 2.6	0.44	1.00	0.61
Brainstem						
*D*_max⁡_ (Gy)	47.7 ± 3.3	48.1 ± 3.5	47.6 ± 3.1	1.00	1.00	1.00
Optical nerve						
*D*_max⁡_ (Gy)	34.7 ± 18.3	37.8 ± 19.3	37.8 ± 17.7	0.58	0.33	1.00
Optic chiasm						
*D*_max⁡_ (Gy)	40.2 ± 15.5	40.8 ± 17.8	42.3 ± 16.3	1.00	0.98	0.35
TMJ						
*D*_2%_ (Gy)	56.6 ± 7.1	53.9 ± 9.1	53.5 ± 8.0	0.24	0.11	1.00
Mean (Gy)	42.9 ± 7.1	39.3 ± 7.7	37.4 ± 7.7	0.02	0.00	0.34
Mandible						
*D*_2%_ (Gy)	67.2 ± 2.1	64.7 ± 2.6	63.7 ± 1.7	0.00	0.00	0.06
Mean (Gy)	52.4 ± 2.1	49.0 ± 1.3	50.2 ± 1.8	0.00	0.03	0.04
Temporal lobe						
*D*_2%_ (Gy)	54.2 ± 7.9	50.8 ± 10.2	50.0 ± 8.3	0.23	0.09	0.92
Mean (Gy)	24.6 ± 8.3	21.4 ± 6.9	21.8 ± 6.6	0.00	0.01	0.31
Parotid R						
Mean (Gy)	31.2 ± 1.9	29.5 ± 2.4	30.4 ± 2.8	1.14	1.00	0.04
*V*_30_	33.6 ± 9.7	35.7 ± 7.7	34.7 ± 9.7	1.00	1.00	1.00
Parotid L						
Mean (Gy)	31.7 ± 2.1	30.1 ± 3.1	31.0 ± 4.3	0.20	1.00	0.28
*V*_30_	37.3 ± 9.6	37.1 ± 9.1	36.1 ± 14.7	1.00	1.00	1.00
Lens						
*D*_max⁡_ (Gy)	8.0 ± 3.4	6.5 ± 1.9	7.4 ± 2.3	0.05	0.57	0.00
Eyes						
*D*_max⁡_ (Gy)	34.0 ± 4.6	27.9 ± 11.4	29.1 ± 10.7	0.06	0.24	0.35
Mean (Gy)	10.5 ± 5.1	10.3 ± 5.1	10.3 ± 4.7	1.00	1.00	1.00
Unspecified tissue						
*D*_1%_ (Gy)	64.7 ± 2.2	61.5 ± 2.9	61.1 ± 2.4	0.00	0.00	0.79
Mean (Gy)	17.7 ± 3.9	16.5 ± 3.5	17.0 ± 3.8	0.00	0.02	0.01

TMJ = temporomandibular joint.

*p*_*α*_: 9F-IMRT versus 2ARC-VMAT; *p*_*β*_: 9F-IMRT versus Hybrid; *p*_*γ*_: 2ARC-VMAT versus Hybrid.

**Table 3:** Comparison of delivery time and MUs for 9F-IMRT, 2ARC-VMAT, and Hybrid IMRT/VMAT plans.

	9F-IMRT	2ARC-VMAT	Hybrid	Pairwise comparison		
				*p*_*α*_	*p*_*β*_	*p*_*γ*_
Delivery time (s)	812 ± 42	179 ± 0	408 ± 10	0.00	0.00	0.00
MU	2256 ± 219	507 ± 43	1394 ± 117	0.00	0.00	0.00

MUs = monitor units.

*p*_*α*_: 9F-IMRT versus 2ARC-VMAT; *p*_*β*_: 9F-IMRT versus Hybrid; *p*_*γ*_: 2ARC-VMAT versus Hybrid.
